# Chemical Kinetics of Hydrogen Atom Abstraction from Propargyl Sites by Hydrogen and Hydroxy Radicals

**DOI:** 10.3390/ijms20133227

**Published:** 2019-06-30

**Authors:** Quan-De Wang, Yanjin Sun, Mao-Mao Sun, Jin-Hu Liang

**Affiliations:** 1 Low Carbon Energy Institute, School of Chemical Engineering, Jiangsu Province Engineering Laboratory of High Efficient Energy Storage Technology and Equipments, China University of Mining and Technology, Xuzhou 221008, China; 2 Combustion Chemistry Centre, School of Chemistry, Ryan Institute, National University of Ireland, Galway H91 TK33, Ireland; 3 School of Environment and Safety Engineering, North University of China, Taiyuan 030051, China

**Keywords:** alkynes, propargyl, abstraction reactions, transition state theory

## Abstract

Hydrogen atom abstraction from propargyl C-H sites of alkynes plays a critical role in determining the reactivity of alkyne molecules and understanding the formation of soot precursors. This work reports a systematic theoretical study on the reaction mechanisms and rate constants for hydrogen abstraction reactions by hydrogen and hydroxy radicals from a series of alkyne molecules with different structural propargyl C-H atoms. Geometry optimizations and frequency calculations for all species are performed at M06-2X/cc-pVTZ level of theory and the hindered internal rotations are also treated at this level. The high-level W1BD and CCSD(T)/CBS theoretical calculations are used as a benchmark for a series of DFT calculations toward the selection of accurate DFT functionals for large reaction systems in this work. Based on the quantum chemistry calculations, rate constants are computed using the canonical transition state theory with tunneling correction and the treatment of internal rotations. The effects of the structure and reaction site on the energy barriers and rate constants are examined systematically. To the best of our knowledge, this work provides the first systematic study for one of the key initiation abstraction reactions for compounds containing propargyl hydrogen atoms.

## 1. Introduction

Alkynes are important intermediates during the oxidation of hydrocarbon fuels, and they are critical initiation species towards soot formation [1,2]. The development of a detailed combustion mechanism to predict the combustion properties of alkynes and soot formation requires a better understanding of the reactivity of alkyne molecules, especially the alkyne molecules with phenyl group. In combustion reaction systems, hydrogen (H) and hydroxy (OH) radicals are usually the most abundant radicals [3,4], and the hydrogen abstraction reactions of alkynes by H and OH radicals play a critical role in understanding the reactivity of alkyne molecules among various reaction pathways. In addition, the activation of alkyne molecules is also a challenging area in organic chemistry [5]. A better understanding of the reactivity of these molecules based on theoretical chemical calculations is also fundamental to organic chemistry.

For alkyne molecules, H-atom abstraction from the acetylenic sites can be ignored due to the large bond dissociation energies (BDEs) [6]. However, abstraction from the propargyl C-H sites forming resonance-stabilized propargyl radicals is believed to be an important step in the formation of large Polycyclic Aromatic Hydrocarbons (PAHs) and soot [2,4,7,8,9,10]. Hence, understanding the underlying chemistry and obtaining accurate rate constants for these abstraction reactions are fundamental to understand the combustion properties of alkynes and soot formation and develop reliable detailed combustion mechanisms. However, limited research has been carried out for the abstraction reactions of alkynes and most studies for alkynes are still limited to propyne.

Miller et al. analyzed the reaction of H with propyne based on a C_3_H_5_ potential and found that the abstraction reaction channel at the propargyl C-H site is also important, especially at high temperatures [11]. They also compared the computed results with experiment by Bentz et al. [12] and good agreement was obtained. Rosado-Reyes et al. studied the thermal reaction of H atoms with propyne in a heated single pulse-shock tube and derived rate constants for H-atom attack on propyne [13]. Hansen et al. [14] incorporated the rate constants obtained by Miller et al. [11] to understand the isomer-specific combustion chemistry in allene and propyne flames and proposed that implementing the accurate theoretical results for important reactions is critical for an accurate prediction of combustion properties. Zádor and Miller used the KinBot software to explore the potential energy surfaces for OH + allene and OH + propyne reactions systematically [15]. It was demonstrated that the major channel of these bimolecular reactions at high temperatures is the abstraction reactions to the formation of propargyl and H_2_O, also indicating the studied reaction systems are very important for soot formation at high temperatures. In addition, Senosiain et al. performed a theoretical study of diacetylene with OH radicals and found that direct abstraction reaction channels for alkyne molecules are very important [16]. Besides these typical studies on allene/propyne and diacetylene, few studies have been performed on larger alkyne molecules. Systematic studies on structure and reaction site effect on energy barriers and rate constants for the H-atom abstraction reactions at the propargyl sites of alkynes are badly needed to develop accurate detailed combustion mechanisms.

Based on the above considerations, in this study, we employ high-level ab initio methods together with transition state theory to study the hydrogen abstraction reactions by H and OH radicals for a series of alkynes with different structural propargyl C-H sites. To obtain accurate reaction barriers and enthalpies for large reaction systems studied in this work, the high-level W1BD [17] and CCSD(T)/CBS [18] methods are used as benchmark methods for small systems to select a proper DFT method for large reactions. The rate constants are computed based on quantum chemistry calculations. The structure and reaction site effect on the energies and rate constants for the abstraction reactions at propargyl C-H sites are systematically investigated.

## 2. Results and Discussion

To investigate the structure and reaction site effect on the abstraction reactions from propargyl C-H sites, eight alkyne molecules with different propargyl C-H sites are considered. The studied alkyne molecules are propyne, 1-butyne, 1-pentyne, penta-1,4-diyne, 4-penten-1-yne, isopentyne, 3-phenyl-1-propyne, and but-3-yn-2-ylbenzene, which can represent the primary, secondary, tertiary, and doubly secondary propargyl H atoms of alkynes and the effect of phenyl substituents which is important for soot precursor formation is also considered.

### 2.1. Geometry Analysis

**Figure 1:**
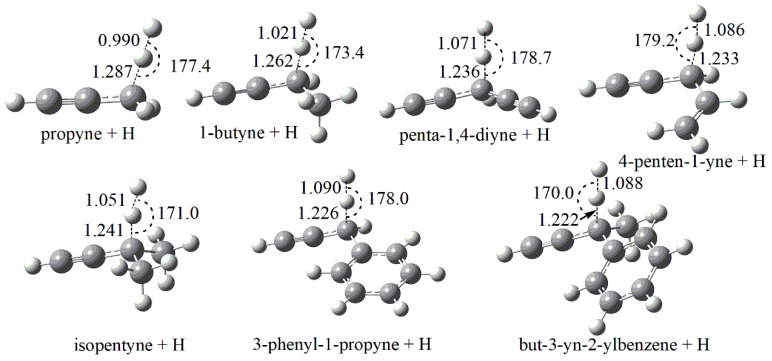
Optimized geometries (in Å and degrees) of TSs for the abstraction reactions by H radical at M06-2X/cc-pVTZ level. The geometry centers of TS for 1-pentyne are identical to that of 1-butyne, and thus not explicitly shown.

**Figure 2:**
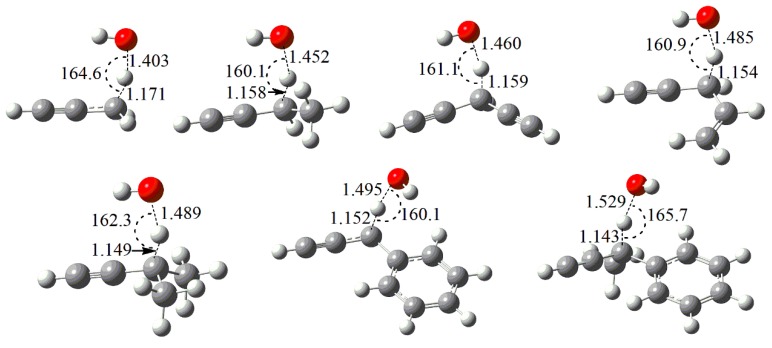
Optimized geometries (in Å and degrees) of TSs for the studied abstraction reactions by OH radical at M06-2X/cc-pVTZ level. The geometry centers of TS for 1-pentyne are identical to that of 1-butyne, and thus not explicitly shown.

Figure 1 and Figure 2 depict the optimized transition state structures at M06-2X/cc-pVTZ level of theory. It should be noted that the lowest energy conformation for each species is obtained by scanning all the relevant internal rotational degrees of freedom and then the global minimum point is used for optimization. As shown in Figure 1, the C-H bond breaking along the reaction coordinate are elongated from an averaged value of 1.09 Å to an averaged value of 1.24 Å and the angles of C-H-H between the breaking C-H bond and the forming H-H bond are near-linear.

Unlike the simple TS structures for H-atom abstraction reactions of alkynes by H atoms at the propargyl reaction sites, the TS structures of abstraction reactions by OH radical are more complex due to the interaction of oxygen atom with other functional groups in the reactant molecules. For the studied reactants of propyne, 1-butyne, 1-pentyne, penta-1,4-diyne, 4-penten-1-yne, and isopentyne, the OH radical is nearly parallel along the C≡C triplet bond of the reactants. However, for 3-phenyl-1-propyne and but-3-yn-2-ylbenzene, the OH radical tends to locate above the benzene ring and the OH radical is nearly parallel along the C-C bond connecting the phenyl group.

**Figure 3:**
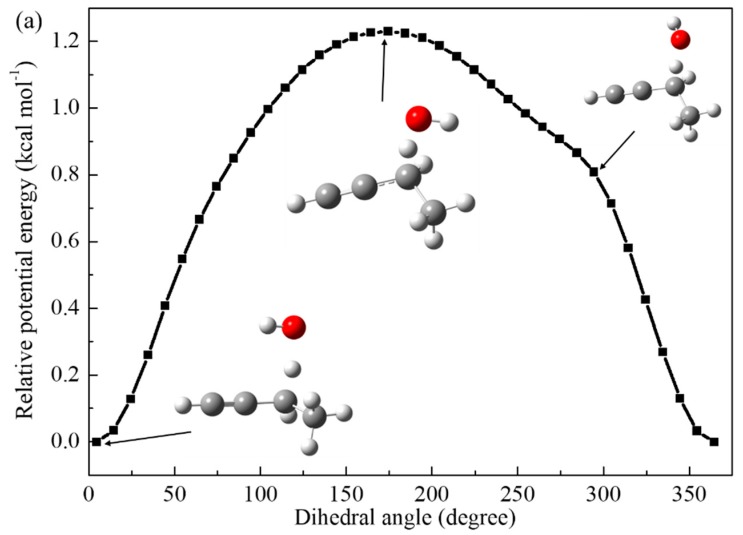

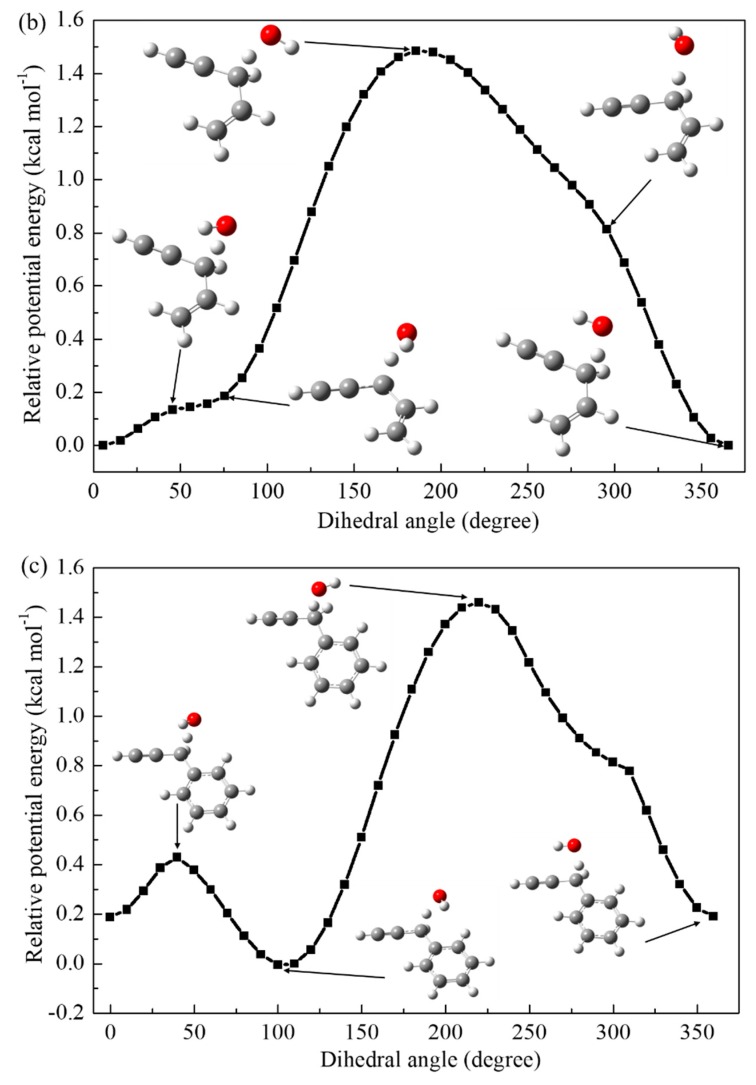
Relative potential energies of the internal rotation of H atom in OH radical along the reaction center for 1-butyne (**a**), 4-penten-1-yne (**b**), and 3-phenyl-1-propyne (**c**), respectively.

**Figure 4:**
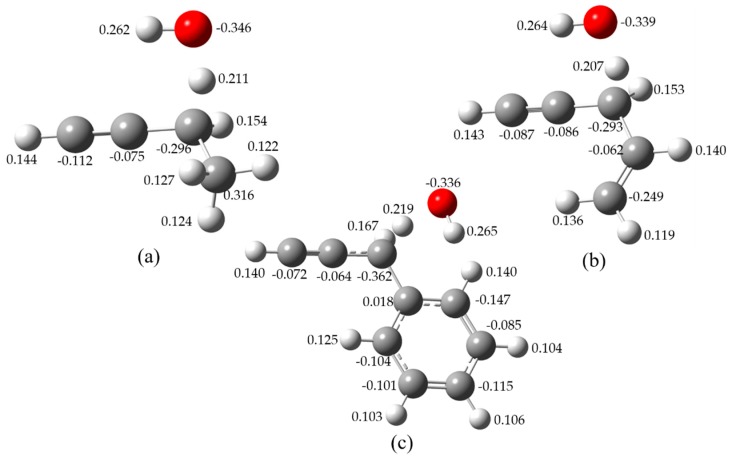
Mulliken charge distributions of the TSs for 1-butyne (**a**), 4-penten-1-yne (**b**), and 3-phenyl-1-propyne (**c**) with OH, respectively.

To further confirm the minimal structures corresponding to the global minima, Figure 3 shows the hindrance potential of relaxed scans of the internal rotation of H atom in OH radical for 1-butyne, 4-penten-1-yne, and 3-phenyl-1-propyne. Figure 4 illustrates the Mulliken charge distributions [19] of the TSs for these three reactions. The relative energies between the highest and lowest structures are computed at the CCSD(T)/cc-pVTZ level of theory to validate the scanned results at the M06-2X/cc-pVTZ level of theory. It has been verified that the two theoretical methods can give similar energy curves and the deviations between CCSD(T)/cc-pVTZ and M06-2X/cc-pVTZ are within 0.5 kcal mol^−1^.

As shown in Figure 4, for the TS of H-abstraction reaction of 1-butyne by OH radical, the energy difference between the lowest and highest energy conformations is 1.23 kcal mol^−1^. The negative charge distributions of the two carbon atoms in the C≡C triplet bond attract the H atom in OH radical pointing to the C≡C triplet bond resulting in the O–H bond parallel to the C≡C triplet bond. The energy difference between the lowest and highest energy conformations for 4-penten-1-yne and 3-phenyl-1-propyne are 1.48 and 1.46 kcal mol^−1^, respectively. The charge distribution of the C atom near the propargyl site in the C=C double bond (−0.062) is slightly large than that in the C≡C triplet bond (−0.086), which makes the OH radical toward parallel to the C≡C triplet bond. However, the strong negative charge distributions of phenyl group in 3-phenyl-1-propyne attract the OH radical shifting toward the aromatic ring to form a more stable TS structure.

From Figure 3, the most unstable TS structures of these three reactants are found when the H atom in OH radical shifts toward the opposite sites along the C≡C triplet bond. Similar trend is observed for the other reaction systems. Based on the above analysis, it can be concluded that the formation of the lowest energy conformations of the TSs for the studied reaction systems are mainly affected through the electron-withdrawing inductive effect by the surrounding groups around the reactivity center. Specifically, the lone pair of electrons of oxygen atom are attracted by the conjugated π-system of phenyl group which is similar to the mesomeric effect. Since the electron-withdrawing inductive effect (-*I* effect) of phenyl group is stronger than C=C double bond and C≡C triplet bond, the OH radical prefers to shift toward the aromatic ring to form a more stable TS structure. In addition, the delocalization effect of the C=C double bond of 4-penten-1-yne is less competitive compared with the C≡C triplet bond, the TS structure of the former is expected to be less stable. Furthermore, due to the interaction between the reactants and OH radical, reactant complexes (RCs) and product complexes (PCs) are formed via weakly hydrogen bond and IRC analysis results are adopted to determine the geometries of these complexes. The optimized geometries are provided as Supplementary Materials.

### 2.2. Reaction Barriers and Enthalpies

The accuracy of the computed rate constants critically depends on the reliability of reaction barriers; thus, it is important to obtain reliable single-point energy results by employing high-level quantum chemistry methods. However, it is computationally prohibitive for the studied large reaction systems. Hence, we have adopted the accurate W1BD and CCSD(T)/CBS methods to benchmark a series of DFT functionals to select an appropriate DFT method, since different DFT functionals provide very different results for various chemical properties.

**Figure 5:**
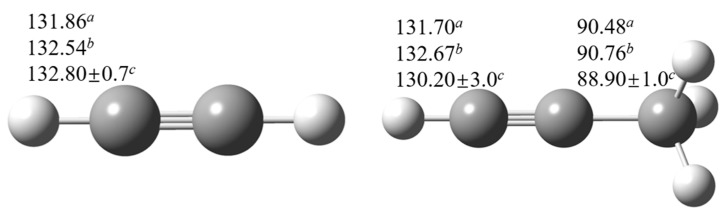
Computed BDEs (in kcal mol^−1^) for C-H bond in acetylene and propyne. aW1BD method, bCCSD(T)/CBS method, cRecommended value from Luo [6].

To verify the selected benchmark methods, the bond dissociation energies (BDEs) of acetylenic C-H bond and propargyl C-H bond for acetylene and propyne are computed first using W1BD and CCSD(T)/CBS methods as shown in Figure 5. It can be seen that the computed BDEs for acetylenic C-H and propargyl C-H bonds by using the W1BD and CCSD(T) methods are in very good correlation with the recommended values by Luo et al. [6], indicating that the W1BD and CCSD(T)/CBS methods should be as accurate as benchmark methods. The BDE of propargyl C-H bond is lower than that of acetylenic C-H bond by 40 kcal mol^−1^, thus, abstraction reactions at the acetylenic site are very difficult. Hence, in this work, we just focus on the hydrogen abstraction channels at the propargyl C-H sites, and systematically investigate the reaction kinetics of structural variant effect on propargyl C-H reaction sites.

Table 1 lists the computed reaction barriers by using high-level W1BD and CCSD(T)/CBS methods and different DFT methods with cc-pVTZ basis set for H-atom abstraction reactions of propyne and 1-butyne by H and OH radicals. The last column represents the absolute average errors compared with the benchmark results from W1BD method. From Table 1 it can be seen that the results via CCSD(T)/CBS method are in good correlation with those from accurate W1BD method. The absolute average error is 0.23 kcal mol^−1^, which is also in good accordance with the computed BDEs shown in Figure 3, indicating that the two high-level methods can provide consistent reliable energy information for the studied reactions. Among the selected 13 DFT functionals, the M08-HX exhibits the best performance and the absolute average errors compared with W1BD and CCSD(T)/CBS methods are 0.36 and 0.24 kcal mol^−1^, respectively. The BMK functional shows good performance for abstraction reactions by H radical, but not for abstraction reactions by OH radical. Similarly, the MPW1K shows better performance only for H-atom abstraction reactions by OH radical. Thus, one should be better to validate these DFT methods before directly apply them for these reaction systems to obtain energy information. In addition, to check the performance of M08-HX functional for 3-phenyl-1-propyne and but-3-yn-2-ylbenzene which contain phenyl group, the results calculated from M08-HX are compared with the results from CCSD(T)/cc-pVTZ level since CCSD(T)/cc-pVQZ level are still computationally prohibitive for a system with over 10 heavy atoms. For H-atom abstraction reactions of 3-phenyl-1-propyne by H and OH radicals, the computed reaction barriers are 5.14 and −0.22 kcal mol^−1^ via CCSD(T)/cc-pVTZ method, while the results at M08-HX/cc-pVTZ level are 5.11 and −0.50 kcal mol^−1^, indicating that the M08-HX/cc-pVTZ level of theory is able to provide reasonable energy information for the studied reaction systems. In addition, from Table 1, the MUE of M08-HX functional relative to W1BD method is mainly affected by the H-abstraction of propyne with H radical. The uncertainty of rate constants calculated with reaction barriers at M08-HX level are found to be within a factor of 2 compared with those at W1BD method over the studied temperature ranges. What is interesting to note is that a strong correlation of the reaction barriers at W1BD and M08-HX levels are fitted to Δ*E*_W1BD_ = 1.0668 × Δ*E*_M08-HX_ + 0.0682 with an adjusted R-squared value of 0.998. Such a correlation expression further reduces the MUE of the four reactions at M08-HX level to 0.075 kcal mol^−1^ relative to the results via W1BD method. Based on this linear correlation, the M08-HX barrier heights for the studied systems are corrected to approach those calculated at W1BD level and listed in Table 2 and Table 3.

Table 2 lists the computed reaction barriers and enthalpies at M08-HX/cc-pVTZ level for the studied H-abstraction reactions by H radical. With the use of this method, the computed potential energies for TSs, RCs and PCs of H-abstraction reactions by OH radical are listed in Table 3 which are relative to their reactants, respectively. For the studied abstraction reaction of propyne with H atom, the predicted reaction barrier and enthalpies are compared with those by Bentz et al. [12] at the QCISD(T)/cc-pVTZ//B3LYP/6-31G(d) level of theory. The reaction barrier and enthalpy by M08-HX/cc-pVTZ in this work are 8.23 and −12.07 kcal mol^−1^, which are slightly lower than the results by Bentz et al. [12] by 0.92 and 0.65 kcal mol^−1^, respectively. While the computed barrier at M08-HX/cc-pVTZ level in this work is much closer to the results from Wang et al. by using the composite G3 method [20]. Compared with those obtained at W1BD and CCSD(T)/CBS methods in this work, the predicted barrier with an value of 8.67 kcal mol^−1^ by Miller et al. [11] at the QCISD(T)/inf//B3LYP/6-311++G(d,p) level of theory is quite close to the present estimation, while the reaction barrier by Bentz et al. [12] is higher by 0.19 and 0.35 kcal mol^−1^. The energy barrier and enthalpy for the H-abstraction reaction of propyne by OH are 2.00 and −26.31 kcal mol^−1^ at M08-HX/cc-pVTZ level, which are also in good agreement with the values of 2.00 and −25.16 kcal mol^−1^ which are computed at the UCCSD(T)-F12b/cc-PVQZ-F12 level [15].

From Table 2 and Table 3, it can be seen that the averaged reaction barrier and enthalpy of H-abstraction reactions by OH radical are lower than that by H radical by values of 5.62 and 14.24 kcal mol^−1^, respectively. Hence, the H-abstraction reactions of alkynes by OH radical are kinetically more favored than that by H radical. The structural nature of the alkynes tends to show similar effect on the reaction barriers and enthalpies of H-abstraction reactions by H and OH radicals. For non-aromatic alkynes, the abstraction reaction at the tertiary (3°) propargyl C-H site in isopentyne is the easiest which has the lowest relative reaction barrier of −0.39 kcal mol^−1^, followed by the secondary (2°) propargyl C-H site in 1-butyne, then the primary (1°) propargyl C-H site in propyne is the most difficult. For the two propargyl C-H sites in 3-phenyl-1-propyne and but-3-yn-2-ylbenzene molecules involving phenyl group, the same trend is also observed that the abstraction reaction at tertiary propargyl C-H site in but-3-yn-2-ylbenzene is easier to occur than that at the secondary propargyl C-H site in 3-phenyl-1-propyne. Thus, the trend for reaction barriers of H-abstraction reactions by H and OH radicals is *E*_0K_(3°) < *E*_0K_(2°) < *E*_0K_(1°). Compared with the results of 1-butyne and 1-pentyne, the length of alkyne chains has negligible effect on the H-abstraction reactions by H radical, but it shows moderate effect on the H-abstraction reactions by OH radical. The reaction barriers of H-abstraction reactions by OH radical decrease with the growth of the chain length. For the five secondary propargyl C-H sites in 1-butyne, 1-pentyne, penta-1,4-diyne, 4-penten-1-yne and 3-phenyl-1-propyne, the existences of vinyl group, phenyl group and acetylenic group all increase the reactivity compared with 1-butyne and 1-pentyne by the following order: vinyl group > phenyl group > acetylenic group. On the other hand, this also means that abstraction reaction at the allylic C-H site is easier than that at the benzyl site, which is easier than that at propargyl site considering the reaction sites connected the other same groups. Table 3 also shows that for the studied H-abstraction reactions by OH radical, the weakly van der Waals RCs and PCs are formed at the entrance and exit channels. The energies of RCs are lower than the reactants by 3.97–5.58 kcal mol^−1^, while the PCs are lower than the products by 3.22–4.30 kcal mol^−1^ for the studied reactions.

### 2.3. Rate Constants

For the abstraction reactions by OH radical, rate constants calculations need to consider the formed Van der Waals complexes (RCs and PCs), and two-TS models are usually employed [21,22]. However, a series of previous studies indicated that this model only showed significant effect at low temperatures below 200 K [21,22,23,24,25,26]. For the interested combustion relevant temperature range of 500–2500 K, the rate-limiting step is the inner TSs. Furthermore, the formed RCs are unstable at high temperatures. Hence, the abstraction reactions by H and OH radicals are all computed by using canonical TST with 1-D hindered rotor approximations for the treatment of internal rotations. The Fourier-series are used for fitting the scanned results of international rotations. To obtain rate constants with minimal uncertainty, the corrected M08-HX barrier height are adopted.

**Figure 6:**
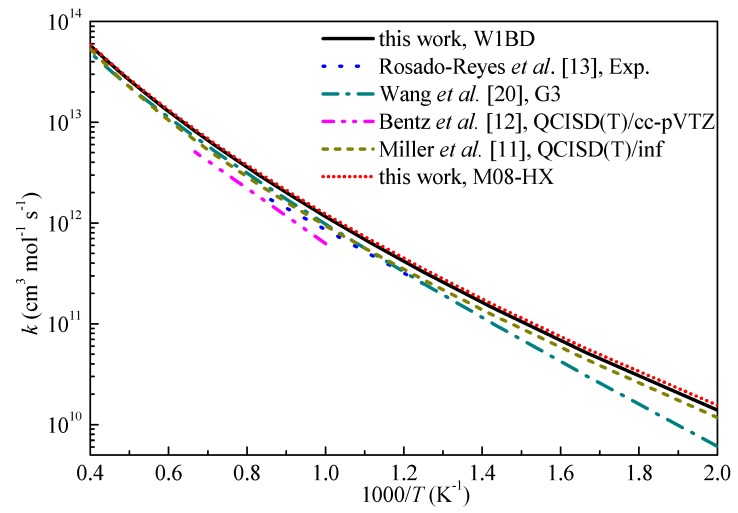
Computed rate constants for the H-abstraction reaction of propyne with H at propargyl site.

**Figure 7:**
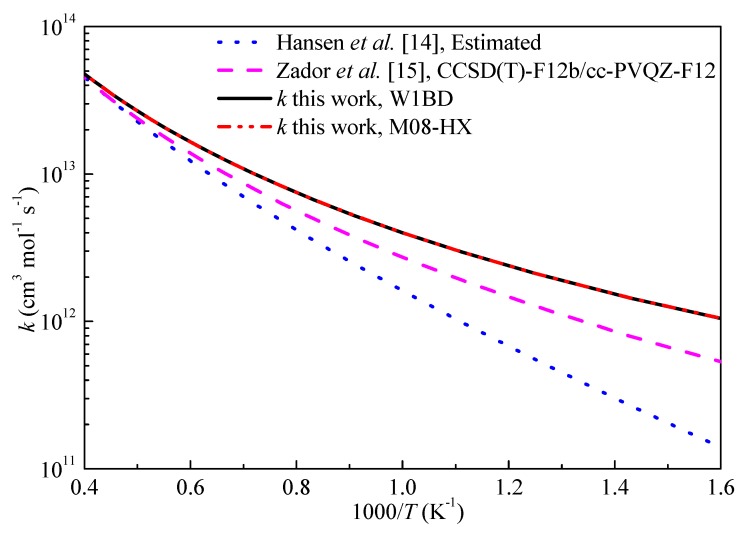
Computed rate constants for the H-abstraction reaction of propyne with OH at propargyl site.

The high-pressure limiting rate constants (*k*_∞_) of H-abstraction reaction of propyne by H and OH radicals in this work are compared with available literature data and shown in Figure 6 and Figure 7, respectively. From Figure 6, the rate constants obtained in this work are in good correlation with the results by Miller et al. [11] at the QCISD(T)/inf//B3LYP/6-311++G(d,p) level of theory over the studied temperature ranges. The rate constants by Wang et al. [20] tended to underestimate the rate constants at low temperature. The predicted results show good agreement with the rate constants derived from a heated single pulse-shock tube at temperatures of 874–1196 K by Rosado-Reyes et al. [13]. For the H-abstraction reaction of propyne with OH, Hansen et al. [14] esimated the rate constants for this reaction and used in kinetic modeling. The deviations of the rate constants between present results and Zador’s calculations [15] are within a factor of 2 and decrease as temperature increasing. The H-abstraction reaction of propyne by OH radical is in fact important at temperatures over 1000 K and it can be seen that the three different sets of rate constants for this reaction at the interested temperature ranges are close to each other. Overall, the results computed at M08-HX level are in good agreement with literature data, but can significantly reduce the computational cost to obtain accurate reaction barriers compared with other expensive W1BD or CCSD(T) methods.

**Figure 8:**
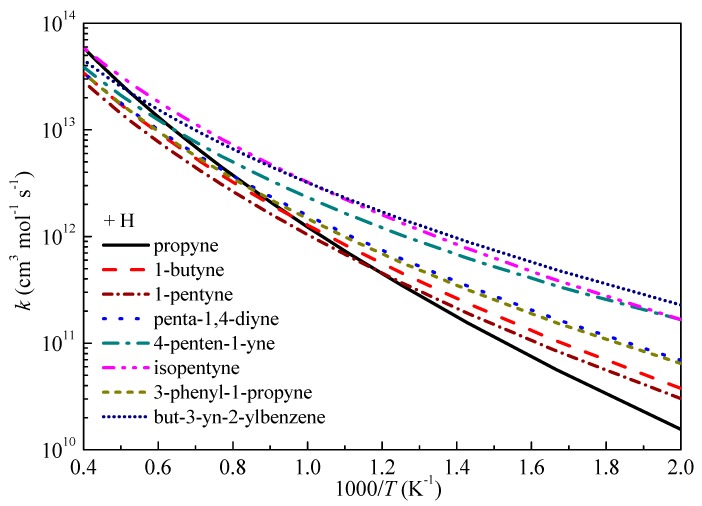
Computed rate constants for the abstraction reactions with H at propargyl site.

Figure 8 compares the rate constants as a function of temperature for the studied H-abstraction reactions by H radical. The TSs for H-abstraction reactions from eight reactants by H and/or OH radicals can be divided into three types: primary propargyl radical (propyne), secondary propargyl radicals (1-butyne, 1-pentyne, penta-1,4-diyne, 4-penten-1-yne and 3-phenyl-1-propyne) and tertiary propargyl radicals (isopentyne and but-3-yn-2-ylbenzene). As can be seen from the graph, the *k*_∞_ of the tertiary propargyl radicals are higher than those of the secondary propargyl radicals. For the five secondary propargyl radicals, in comparison with 1-butyne, the trend is *k*_∞_(1-pentyne) < *k*_∞_(1-butyne) < *k*_∞_(3-phenyl-1-propyne) ≈ *k*_∞_(penta-1,4-diyne) < *k*_∞_(4-penten-1-yne) which suggests the positive (+) or negative (-) influence of different groups on the reaction activity is: –C_2_H_5_ (-) < –CH_3_ (+) < –C_6_H_5_ ≈ –C_2_H < –C_2_H_3_. In addition, the –C_2_H_3_ group tends to show stronger influence on the reaction activity especially at low temperature since the deviation between *k*_∞_(penta-1,4-diyne) and *k*_∞_(4-penten-1-yne) is larger than those between the other secondary propargyl radicals.

The *k*_∞_ of the primary propargyl radical (propyne) tends to show different trend with that of the secondary propargyl radicals which is slower at low temperatures, but overtaking the *k*_∞_ of secondary propargyl radicals at high temperatures. For the tertiary propargyl radicals, *k*_∞_(isopentyne) is slower than that of but-3-yn-2-ylbenzene at temperatures lower than 1000 K which is consistent with the trend of the secondary propargyl radicals that phenyl group has stronger positive influence on the reaction reactivity. However, as the temperature increasing, *k*_∞_(but-3-yn-2-ylbenzene) is overtaken by *k*_∞_(isopentyne).

**Figure 9:**
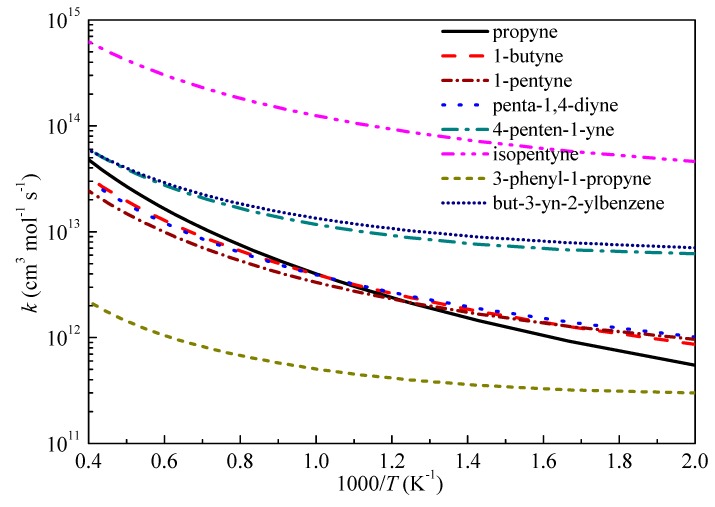
Computed rate constants for the abstraction reactions with OH at propargyl site.

Unlike the H-abstraction reactions of the studied alkyne molecules by H radical, the rate constants by OH radical exhibit large differences. Figure 9 shows that the rate constant of a tertiary propargyl radical (isopentyne) is significantly larger than the others over the whole temperature range, while the *k*_∞_ of a secondary propargyl radical (3-phenyl-1-propyne) is the smallest. It can be clearly seen from this graph that the tertiary propargyl radicals (isopentyne and but-3-yn-2-ylbenzene) have higher *k*_∞_ than secondary propargyl radicals which is consistent with H-abstraction by H atom. The deviations between the primary and secondary propargyl C-H sites with alkyl groups (propyne, 1-butyne and 1-pentyne) are small which are within 3 times over the whole temperature range. For secondary propargyl radicals, (1) the *k*_∞_ of penta-1,4-diyne is similar to that of 1-butyne which may suggests the equivalent influence of methyl and acetylenic group on rate constant calculation for H-abstraction reactions of alkynes by OH radical; (2) the vinyl group in 4-penten-1-yne significantly increases the rate constants compared with *k*_∞_ of 1-butyne. (3) the phenyl group in 3-phenyl-1-propyne significantly decreases the rate constants compared with *k*_∞_ of 1-butyne. Hence, the methyl, vinyl and acetylenic groups in secondary propargyl radicals tends to increase the reactivity of H-abstraction reaction by OH radical compared with the primary propargyl radicals, the trend of influence for these three group are –C_2_H ≈ –CH_3_ < –C_2_H_3_. While phenyl group tend to decrease the reactivity of H-abstraction reaction by OH radical. To facilitate kinetic modeling studies, the computed rate constants are fitted into the modified Arrhenius format as shown in Table 4.

## 3. Computational Methodology

The quantum chemistry calculations in this paper are carried out using Gaussian 09 software [27]. Geometry optimization and frequency analysis are performed at M06-2X/cc-pVTZ level [28]. The resulted frequencies and zero-point energies (ZPEs) are scaled by 0.96 and 0.97, respectively. For the treatment of internal rotations, relaxed potential energy scans are performed as a function of the corresponding dihedral angle with an interval of 10° at M06-2X/cc-pVTZ level. The scanned results are then fitted to a Fourier-series used for rate constant calculations. For the transition state structures, intrinsic reaction coordinate (IRC) calculations [29] are carried out to make sure the saddle points connect the desired reactants and products. Moreover, the IRC results for abstraction reactions by OH radical are also used to determine the structures of the formed reactant complex (RC) and product complex (PC) at the entrance and exit channels.

To obtain reliable rate constants, single-point energy calculations should be performed with high-level quantum chemistry methods. For the studied reaction systems (C3-C10) in this work, high-level CCSD(T)/CBS or very accurate composite W1BD methods can only be used for small systems since the largest molecule (but-3-yn-2-ylbenzene) contains 10 carbon atoms. Considering the trade-off between accuracy and computational cost, W1BD and CCSD(T)/CBS methods are employed for the abstraction reactions of propyne and 1-butyne as benchmark results to choose an appropriate DFT method for larger reaction systems. The T1 diagnostics [30] are complemented to CCSD(T) calculations to check the multi-reference nature of the studied reactions. Generally, a single-reference coupled cluster calculation for closed-shell species is considered to be reliable if the T1 diagnostic value is within 0.020 [30,31]. For open-shell systems, a higher threshold value for the T1 diagnostic up to 0.044 can be acceptable [32]. For all the species during open-shell CCSD(T) calculations, it is found that all T1 diagnostics values are within 0.025, which indicates that single-reference methods are adequate for the studied reactions in this work.

To select an appropriate DFT method, the computed reaction barriers using 13 typical DFT methods including B3LYP [33,34], BH&HLYP [35], B2PLYP [36], B2PLYPD3 [37], CAM-B3LYP [38], BMK [39], M05-2X [40], MN15 [41], MPW1K [42], PBE0 [43], ωB97XD [44], M08HX [45] and M06-2X [28] with cc-pVTZ basis set are compared with the benchmark results via W1BD and CCSD(T)/CBS methods. In addition, basis set effect on the computed results are also examined by using M06-2X functional with cc-pVTZ, cc-pVQZ, aug-cc-pVTZ and aug-cc-pVQZ basis set [46]. It is found that the basis sets show small influence on reaction barriers for propyne and 1-butyne with H and OH radicals and the deviations are less than 0.40 kcal mol^−1^. Thus, all the DFT calculations are performed using cc-pVTZ basis set unless otherwise mentioned.

Based on quantum chemistry calculations, reaction rate constants of the hydrogen abstraction reactions are computed using the canonical transition state theory. The quantum mechanical tunneling corrections are included by using unsymmetrical Eckart barrier model [47] for reactions with positive barrier heights, while the Wigner method [48] is adopted for some H-abstraction reactions by OH radical with slight negative barrier heights. It has been shown that the two methods can provide very close tunneling coefficients under the studied temperature ranges [49,50]. The rate constants are computed at temperatures from 500 to 2500 K in increments of 100 K and are fitted into the modified Arrhenius equation as $k\left(T\right)=AT^{n}\exp\left(-E_{a}/RT\right)$, in which *A* is the Arrhenius prefactor, *E*_a_ is the barrier height, and *n* is the temperature exponent representing the deviation from the standard Arrhenius equation. The fitted rate coefficients listed in Table 4 can be directly used for kinetic modeling studies. The rate constant calculations are performed using Multiwell software [51].

## 4. Conclusions

This work reports a systematic ab initio and chemical kinetic study of H-abstraction reactions from a series of alkyne molecules by H and OH radicals at the propargyl C-H site. Different types of propargyl C-H sites and the effect of methyl, ethyl, vinyl, phenyl and acetylenic groups in the studied alkyne molecules on reaction reactivity are considered. Geometry optimizations for all species are performed at M06-2X/cc-pVTZ level of theory and each optimized geometry is confirmed to be the lowest energy conformation by scanning all the relevant internal rotational degrees of freedom. It is found that the TS structures of the H-abstraction reactions by H radical are similar, while the existence of vinyl, phenyl and acetylenic groups greatly affects the TS structures of the H-abstraction reactions by OH radical due to the electron-withdrawing inductive effect.

To obtain reliable energy information, accurate W1BD and CCSD(T)/CBS methods are used as benchmark for small reaction systems toward the selection of accurate DFT functionals for large reaction systems. The effect of basis set during DFT calculations are considered and it is found that the M08-HX/cc-pVTZ method exhibits better performance among the selected DFT functionals. General trends of the reaction barriers of the H-abstraction reactions from these alkyne molecules by H and OH radicals have been outlined. The computed results reveal that the reaction barriers decrease as the nature of the abstracted H atom at the propargyl C-H site from primary through secondary to tertiary. The existence of vinyl, phenyl and acetylenic groups also decreases the energy barriers with the following order: vinyl > phenyl > acetylenic.

Based on the quantum chemistry calculations, rate constants are computed using the canonical transition state theory with tunneling correction and the treatment of internal rotations. The computed rate constants for abstraction reactions of propyne by H and OH radicals are compared with literature data and show good agreement. The TSs for H-abstraction reactions from eight alkynes by H and/or OH radicals are further divided into three types: primary propargyl, secondary propargyl and tertiary propargyl radicals. The high-pressure limiting rate constants of the tertiary propargyl radicals are consistently faster than those of the secondary propargyl radicals for H-abstraction reactions by H and OH radicals. For the secondary propargyl radicals, the ability of different groups on increasing reaction activity of H-abstraction reactions by H atom is: –C_2_H_5_ (negative) < –CH_3_ < –C_6_H_5_ ≈ –C_2_H < –C_2_H_3_, while the trend for H-abstraction reactions by OH atom is –C_6_H_5_ (negative) < –C_2_H ≈ –CH_3_ < –C_2_H_3_. Finally, the computed rate constants are fitted into the three-parameter Arrhenius expressions to facilitate kinetic modeling studies. Compared with literature studies on small reactions in this work, the employed ab initio calculations together with statistical transition state theory provide an accurate description of the chemical kinetics of the studied abstraction reactions. However, the subsequent reactions of the formed radicals of alkyne molecules can exhibit complex reaction pathways, which may need direct dynamics to uncover [52]. Currently, direct dynamics is limited to small polyatomic systems because it still depends on accurate potential energy surfaces (PES) from ab initio calculations. The benchmark results of DFT methods in this work also provide fundamental information to select efficient method for PES constructions used in future direct dynamics studies on these reaction systems.

## Figures and Tables

**Table 1:** The computed reaction barriers using different methods for H-abstraction reactions of propyne and 1-butyne by H and OH radicals (units: kcal mol^−1^).

Methods		Propyne + H	Propyne + OH	1-Bytyne + H	1-bytyne + OH	MUE ^2^
Benchmark	W1BD	8.96	2.23	6.76	0.80	
	CCSD(T)/CBS	8.80	2.07	6.52	0.43	0.23
DFT ^1^	M06-2X	9.27	1.17	7.19	−0.36	0.74
	B3LYP	2.58	−2.29	0.76	−3.83	5.38
	CAM-B3LYP	4.73	−1.18	2.71	−2.58	3.77
	BH&HLYP	6.47	4.38	4.54	2.74	2.20
	BMK	8.71	0.84	6.78	−0.57	0.76
	M05-2X	10.09	1.32	8.11	−0.18	1.09
	MN15	8.27	0.38	6.31	−1.16	1.24
	M08-HX	8.23	2.03	6.41	0.65	0.36
	MPW1K	6.98	2.54	4.96	1.13	1.11
	B2PLYP	6.30	0.37	4.20	−1.09	2.24
	ωB97XD	7.41	−0.78	5.40	−2.35	2.27
	B2PLYPD3	5.93	−0.30	3.74	−1.90	2.82
	PBE0	4.78	−2.47	2.76	−3.69	4.34

^1^ DFT calculations are performed with cc-pVTZ basis set. ^2^ Mean unsigned error are relative to benchmark values from W1BD method.

**Table 2:** Reaction barriers and enthalpies for different alkynes reactanting with H atoms at M08-HX/cc-pVTZ level (kcal mol^−1^). All the energies are relative to the reactants.

Reactions	Barriers ^1^	Enthalpies	BDE
propyne + H	8.23 (8.85)	−12.07	89.74
	9.15 ^2^	−12.72 ^2^	
	8.40 ^3^	−14.00 ^3^	
	8.67 ^4^	−12.73 ^4^	
1-butyne + H	6.41 (6.91)	−15.56	86.26
1-pentyne + H	6.26 (6.75)	−14.59	87.23
penta-1,4-diyne + H	5.69 (6.14)	−23.27	78.54
4-penten-1-yne + H	4.56 (4.93)	−25.75	76.07
isopentyne + H	5.05 (5.46)	−18.26	83.55
3-phenyl-1-propyne + H	5.11 (5.52)	−23.64	78.17
but-3-yn-2-ylbenzene + H	4.48 (4.85)	−25.87	75.94

^1^ The values in parentheses represent the corrected barriers using linear correlation. ^2^ Results were performed at QCISD(T)/cc-pVTZ//B3LYP/6-31G(d) level of theory by Bentz et al. [12]. ^3^ Results were performed at G3//UB3LYP/6-31G(d) level of theory by Wang et al. [20]. ^4^ Results were computed at QCISD(T)/inf//B3LYP/6-311++G(d,p) level by Miller et al. [11].

**Table 3:** Potential energies for the studied alkynes with OH at M08-HX/cc-pVTZ level (kcal mol^−1^). All the energies are relative to the reactants.

Reactions	Reactants	RC	Barriers ^1^	PC	Products
propyne + OH	0.00	−4.15	2.03 (2.23)	−29.53	−26.31
1-butyne + OH	0.00	−4.40	0.65 (0.76)	−33.63	−29.80
1-pentyne + OH	0.00	−5.11	−0.07 (−0.01)	−33.79	−28.83
penta-1,4-diyne + OH	0.00	−3.97	0.42 (0.52)	−40.99	−37.52
4-penten-1-yne + OH	0.00	−5.58	−0.96 (−0.96)	−43.75	−39.99
isopentyne + OH	0.00	−4.51	−0.39 (−0.35)	−36.54	−32.50
3-phenyl-1-propyne + OH	0.00	−4.62	−0.50 (−0.47)	−41.15	−37.88
but-3-yn-2-ylbenzene + OH	0.00	−4.31	−1.03 (−1.03)	−44.41	−40.11

^1^ The values in parentheses represent the corrected barriers using linear correlation.

**Table 4:** Fitted Arrhenius coefficients of the studied reactions in *k*(*T*) = *AT^n^*exp(-*E_a_*/*RT*). (*A*: cm^3^ mol^−1^ s^−1^; *E_a_*: cal mol^−1^).

Alkyne Molecules	+ H			+ OH		
	*A*	*n*	*E*_a_	*A*	*n*	*E*_a_
propyne	6.40 × 10^7^	1.94	7042	4.71 × 10^5^	2.38	986
1-butyne	1.83 × 10^7^	1.97	4921	4.52 × 10^5^	2.31	−32
1-pentyne	2.41 × 10^7^	1.92	5191	1.78 × 10^5^	2.38	−639
penta-1,4-diyne	1.70 × 10^7^	1.97	4291	2.51 × 10^5^	2.37	−427
4-penten-1-yne	9.31 × 10^6^	2.03	3203	2.45 × 10^5^	2.42	−1959
isopentyne	4.20 × 10^7^	1.91	3856	3.76 × 10^7^	2.10	−1042
3-phenyl-1-propyne	1.03 × 10^7^	2.03	4331	1.04 × 10^4^	2.39	−2354
but-3-yn-2-ylbenzene	3.75 × 10^7^	1.87	3110	1.38 × 10^6^	2.20	−1745

## Supplementary Materials

Supplementary materials can be found at https://www.mdpi.com/1422-0067/20/13/3227/s1.
