# Supplementary material for: Chemical Kinetics of Hydrogen Atom Abstraction from Propargyl Sites by Hydrogen and Hydroxy Radicals
Source: Int J Mol Sci. 2019 Jun 30;20(13):3227. doi: 10.3390/ijms20133227 (PMC6650822; doi:10.3390/ijms20133227)
Supplement: Supplementary file 1 [file ijms-20-03227-s001.pdf]

Chemical Kinetics of Hydrogen Atom Abstraction from Propargyl Sites by Hydrogen and Hydroxy Radicals

Quan-De Wang [1,*], Yanjin Sun [2], Mao-Mao Sun [1] and Jin-Hu Liang [3]

[1] Low Carbon Energy Institute, School of Chemical Engineering, Jiangsu Province Engineering Laboratory of High Efficient Energy Storage Technology and Equipments, China University of Mining and Technology, Xuzhou 221008, People's Republic of China;
[2] Combustion Chemistry Centre, School of Chemistry, Ryan Institute, National University of Ireland, Galway, H91 TK33, Ireland; Y.SUN5@nuigalway.ie
[3] School of Environment and Safety Engineering, North University of China, Taiyuan 030051, People's Republic of China; jhliang@nuc.edu.cn (J.L.)
* Correspondence: quandewang@cumt.edu.cn; Tel.: +86-151-6213-7355 (Q.W.)

List of the optimized geometries for the studied abstraction reactions

Reactants:

propyne

C	0.	0.	1.41612
H	0.	0.	2.47809
C	0.	0.	0.21945
C	0.	0.	-1.23799
H	0.	1.01951	-1.62118
H	0.88292	-0.50976	-1.62118
H	-0.88292	-0.50976	-1.62118

1-butyne

C	1.94504	-0.26274	0.
H	2.93928	-0.63608	0.
C	0.82671	0.16534	0.
C	-0.55049	0.65262	0.
H	-0.69921	1.28713	0.87519
H	-0.69921	1.28713	-0.87519
C	-1.56799	-0.49026	0.
H	-2.58276	-0.09542	0.
H	-1.43888	-1.11629	-0.88098

H	-1.43888	-1.11629	0.88098

1-pentyne

C	-1.16969	0.42558	-0.41072
C	0.08785	0.99275	0.26002
C	1.25896	0.14049	0.06668
C	-1.58638	-0.91685	0.17258
C	2.20336	-0.57445	-0.11076
H	-0.98255	0.32632	-1.48079
H	-1.97484	1.15217	-0.29215
H	0.30785	1.986	-0.1345
H	-0.09015	1.10931	1.33187
H	-2.48235	-1.29722	-0.31598
H	-0.79229	-1.65305	0.04941
H	-1.79682	-0.82687	1.2395
H	3.04654	-1.20179	-0.26416

penta-1,4-diyne

C	-2.22464	-0.57609	-0.00009
C	-1.22099	0.07296	0.00013
C	0.	0.88123	-0.00007
C	1.22101	0.073	0.00008
C	2.22463	-0.57609	0.00013
H	-3.11339	-1.15788	0.00011
H	0.00001	1.53303	0.876
H	-0.00002	1.53268	-0.87641
H	3.11334	-1.15794	-0.00075

4-penten-1-yne

C	2.3828	-0.44243	-0.14662
C	1.33029	0.11817	-0.04403
C	0.03236	0.77862	0.09501
C	-1.0692	-0.19865	0.41035

C	-2.17852	-0.31388	-0.29879
H	3.31812	-0.93655	-0.24235
H	-0.2039	1.32088	-0.82176
H	0.10716	1.51912	0.89613
H	-0.90606	-0.82838	1.27777
H	-2.35364	0.29795	-1.17556
H	-2.94807	-1.02397	-0.02972

isopentyne

C	2.21411	0.	-0.12131
H	3.25352	0.	-0.33964
C	1.04317	0.	0.13166
C	-0.39502	0.	0.4085
H	-0.51938	0.	1.49433
C	-1.05101	-1.26331	-0.15562
H	-2.11446	-1.27048	0.08329
H	-0.93993	-1.28979	-1.23966
H	-0.59336	-2.16082	0.25616
C	-1.05101	1.26331	-0.15562
H	-0.93993	1.28979	-1.23966
H	-2.11447	1.27048	0.08328
H	-0.59337	2.16082	0.25616

3-phenyl-1-propyne

C	-3.47553	-0.68064	-0.00093
H	-4.28697	-1.36627	-0.00348
C	-2.56655	0.09802	-0.00033
C	-1.45927	1.04732	0.00114
C	1.0302	1.27285	-0.0004
C	2.31597	0.75899	-0.0008
C	2.51469	-0.6162	-0.00037
C	1.41945	-1.46447	0.00044

C	0.1295	-0.94681	0.00084
C	-0.07514	0.42518	0.00044
H	0.87808	2.3459	-0.00079
H	3.16402	1.43032	-0.00148
H	3.51758	-1.02056	-0.0007
H	1.56465	-2.53634	0.00075
H	-0.72376	-1.61148	0.00141
H	-1.55691	1.69523	0.87555
H	-1.55663	1.69778	-0.87138

but-3-yn-2-ylbenzene

C	3.06227	1.36943	-0.51158
H	3.74895	2.17294	-0.61802
C	2.28792	0.4623	-0.40458
C	1.36055	-0.65769	-0.23917
C	-1.08781	-1.095	-0.56829
C	-2.42618	-0.75305	-0.44947
C	-2.78319	0.47682	0.08582
C	-1.79411	1.35804	0.49702
C	-0.45489	1.01411	0.37499
C	-0.09031	-0.21623	-0.15771
H	-0.81133	-2.05488	-0.98898
H	-3.18993	-1.44452	-0.77876
H	-3.82605	0.74793	0.17681
H	-2.06444	2.32031	0.91049
H	0.31596	1.70818	0.68412
H	1.46406	-1.30055	-1.1168
C	1.73942	-1.47902	1.00578
H	1.07229	-2.33468	1.10103
H	2.76708	-1.83118	0.9352
H	1.64137	-0.86189	1.89801

Transition states of reactants with H:

propyne + H

C	-1.4976	0.09869	0.
H	-2.54849	0.25299	0.
C	-0.31215	-0.08914	0.
C	1.10592	-0.26182	0.
H	1.65019	0.90419	0.
H	1.50636	-0.71257	0.90312
H	1.50636	-0.71256	-0.90313
H	2.10854	1.78158	0.

1-butyne + H

C	2.0005	-0.2258	-0.10257
H	3.01236	-0.49858	-0.27584
C	0.85864	0.08105	0.10334
C	-0.51256	0.45476	0.30493
H	-0.72098	1.45845	-0.43135
H	-0.6872	0.86882	1.2962
H	-0.99777	2.22716	-1.04416
C	-1.54577	-0.57091	-0.11799
H	-2.54906	-0.15729	-0.02927
H	-1.38169	-0.87268	-1.15084
H	-1.48055	-1.46045	0.50902

1-pentyne + H

C	-2.22978	-0.57726	-0.03969
H	-3.09086	-1.19925	-0.04513
C	-1.26166	0.1327	-0.04106
C	-0.0924	0.96453	-0.0079
H	0.13166	1.22411	1.20789
H	-0.27852	1.95451	-0.42018

H	0.45815	1.42109	2.1567
C	1.20146	0.33288	-0.4961
H	1.12455	0.16243	-1.5728
H	2.00596	1.05439	-0.34504
C	1.52276	-0.97174	0.21914
H	1.60351	-0.8079	1.29484
H	0.73803	-1.70835	0.05058
H	2.46519	-1.38771	-0.13323

penta-1,4-diyne + H

C	-2.25144	-0.60302	0.10778
H	-3.15936	-1.13243	0.26291
C	-1.2236	-0.01654	-0.0735
C	-0.00003	0.72575	-0.26683
H	0.00001	1.62908	0.57633
H	-0.00012	1.28745	-1.20011
H	0.00032	2.42883	1.28831
C	1.22362	-0.01655	-0.07367
C	2.25142	-0.60304	0.10783
H	3.15934	-1.1325	0.26292

4-penten-1-yne + H

C	-2.12339	-0.59444	0.02513
H	-2.99123	-1.19972	0.12091
C	-1.14873	0.09312	-0.09265
C	0.02245	0.92506	-0.19909
H	-0.0141	1.69593	0.76237
H	-0.04843	1.6281	-1.02847
C	1.34229	0.24528	-0.13322
C	1.52402	-1.04098	0.12911
H	2.51764	-1.46338	0.1775
H	2.19707	0.89122	-0.29735

H	0.68412	-1.70358	0.29422
H	-0.04487	2.38322	1.59508

isopentyne + H

C	2.2611	0.00007	-0.09154
H	3.31563	0.00014	-0.21926
C	1.06939	-0.00003	0.0497
C	-0.36002	-0.00002	0.23813
H	-0.53063	-0.00018	1.46709
H	-0.83675	-0.00031	2.47274
C	-1.04078	1.2736	-0.23133
H	-2.08724	1.26963	0.0738
H	-0.55554	2.1539	0.18541
H	-0.99858	1.34151	-1.31993
C	-1.04066	-1.27358	-0.23142
H	-2.08733	-1.26932	0.07307
H	-0.99784	-1.34171	-1.31996
H	-0.55588	-2.15391	0.18578

3-phenyl-1-propyne + H

C	-3.45243	-0.74276	-0.26125
H	-4.26564	-1.41457	-0.38805
C	-2.53683	0.01982	-0.13103
C	-1.44388	0.93808	0.06523
C	1.01348	1.25385	-0.17296
C	2.31397	0.77768	-0.19025
C	2.55952	-0.57948	-0.0279
C	1.49649	-1.45294	0.15105
C	0.19413	-0.97645	0.16894
C	-0.05916	0.38187	0.00463
H	0.8231	2.31269	-0.29987
H	3.13662	1.465	-0.33309

H	3.57396	-0.95346	-0.04336
H	1.68093	-2.51139	0.27509
H	-0.63547	-1.65807	0.30189
H	-1.58458	1.38846	1.19657
H	-1.55549	1.83559	-0.5415
H	-1.68515	1.81774	2.19356

but-3-yn-2-ylbenzene + H

C	3.05221	1.49203	0.25821
H	3.75026	2.29203	0.29799
C	2.26315	0.59004	0.21507
C	1.34472	-0.52385	0.17316
C	-1.06907	-1.14588	0.31313
C	-2.41981	-0.852	0.23549
C	-2.83506	0.43868	-0.06723
C	-1.88962	1.42925	-0.28178
C	-0.53517	1.13584	-0.19775
C	-0.11137	-0.15667	0.09134
H	-0.75133	-2.15054	0.56493
H	-3.15018	-1.62888	0.41627
H	-3.88964	0.66965	-0.12902
H	-2.20447	2.43776	-0.51334
H	0.20074	1.91187	-0.35913
H	1.45637	-1.07384	1.2586
H	1.47612	-1.71302	2.13831
C	1.77749	-1.6002	-0.81891
H	1.69603	-1.21865	-1.83684
H	2.81006	-1.89119	-0.63526
H	1.14126	-2.4786	-0.7268

Transition states of reactants with OH:

propyne + OH

C	1.90851	-0.62521	0.
H	2.72593	-1.30375	0.00001
C	0.99843	0.15523	-0.00001
C	-0.12327	1.06077	0.
H	-1.11739	0.44164	0.00004
H	-0.16821	1.67319	0.89777
H	-0.16825	1.67316	-0.89779
O	-2.06866	-0.5891	0.
H	-1.42478	-1.31615	-0.00002

1-butyne + OH

C	-2.12759	-0.6381	-0.17724
H	-3.04494	-1.08443	-0.47376
C	-1.1012	-0.12422	0.16946
C	0.15811	0.48259	0.55002
H	0.97641	-0.32981	0.44561
H	0.16199	0.72813	1.61248
O	1.73777	-1.43869	-0.10041
H	0.96243	-1.90347	-0.45602
C	0.5732	1.65606	-0.32472
H	-0.15696	2.46198	-0.25289
H	1.54422	2.03435	-0.01195
H	0.6396	1.34475	-1.36528

1-pentyne + OH

C	2.30365	-0.59155	-0.42814
H	3.18674	-0.84016	-0.96396
C	1.31355	-0.3198	0.19165
C	0.10113	0.01993	0.90928
H	-0.19172	1.09675	0.5999
H	0.2985	0.09666	1.97902

O	-0.39572	2.30751	-0.19175
H	0.32368	2.11582	-0.81606
C	-1.09277	-0.87919	0.59893
H	-0.85542	-1.89352	0.92815
H	-1.93648	-0.53627	1.19831
C	-1.45999	-0.87619	-0.878
H	-2.32586	-1.50913	-1.06557
H	-1.6955	0.13655	-1.20572
H	-0.63166	-1.24602	-1.48234

penta-1,4-diyne + OH

C	1.63774	-1.7242	-0.26021
H	2.34113	-2.43744	-0.61453
C	0.8332	-0.93648	0.14533
C	-0.11352	0.05616	0.62706
H	0.28196	1.09898	0.31193
H	-0.1159	0.09918	1.71699
O	1.15796	2.14533	-0.20833
H	1.77911	1.55109	-0.66163
C	-1.45671	-0.08518	0.09866
C	-2.56697	-0.1783	-0.33772
H	-3.55243	-0.26651	-0.72486

4-penten-1-yne + OH

C	-0.54007	2.20068	-0.11224
H	-0.74095	3.19121	-0.4402
C	-0.3076	1.09036	0.27345
C	-0.04943	-0.26769	0.71066
H	-0.89651	-0.93159	0.29328
H	-0.18201	-0.35809	1.79044
O	-2.13571	-1.3485	-0.41178
H	-2.30794	-0.47816	-0.80782

C	1.24417	-0.86492	0.2624
C	2.11917	-0.27735	-0.53909
H	3.03329	-0.77795	-0.82539
H	1.43481	-1.8636	0.6371
H	1.94759	0.71959	-0.92434

isopentyne + OH

C	-0.00083	-0.0038	-0.00012
H	-0.00144	0.00355	1.06228
C	0.00614	-0.00016	-1.19927
C	0.00619	-0.01181	-2.65471
H	-0.50642	-0.99345	-2.9607
O	-1.1938	-2.31317	-2.9015
H	-1.20958	-2.3381	-1.93035
C	-0.83921	1.11399	-3.23934
H	-0.40008	2.07957	-2.98333
H	-0.87667	1.026	-4.3243
H	-1.85402	1.08248	-2.84843
C	1.41692	-0.06712	-3.22975
H	1.37333	-0.15161	-4.31475
H	1.95864	0.84464	-2.97296
H	1.96644	-0.91788	-2.8324

3-phenyl-1-propyne + OH

C	-3.1296	-1.57418	-0.00939
H	-3.84224	-2.32781	0.22105
C	-2.33258	-0.72005	-0.27529
C	-1.37522	0.31918	-0.58358
C	1.02973	0.96726	-0.62759
C	2.37144	0.73453	-0.37793
C	2.77405	-0.46477	0.19789
C	1.82607	-1.42261	0.52118

C	0.47943	-1.18916	0.27242
C	0.07133	0.00563	-0.30638
H	0.71104	1.90473	-1.06763
H	3.10474	1.48738	-0.63318
H	3.82169	-0.64881	0.39227
H	2.13254	-2.35819	0.96894
H	-0.26012	-1.93775	0.52273
H	-1.65764	1.24745	0.03816
H	-1.5065	0.67223	-1.60801
O	-1.50916	2.48152	0.8694
H	-0.71811	2.17363	1.34249

but-3-yn-2-ylbenzene + OH

C	2.6944	-1.93482	-0.58363
H	3.28576	-2.75085	-0.92024
C	2.03043	-1.01005	-0.20935
C	1.24972	0.1195	0.26488
C	-0.83623	-1.2873	-0.10243
C	-2.21682	-1.40043	-0.20401
C	-3.01991	-0.27474	-0.11047
C	-2.43592	0.97049	0.08553
C	-1.05937	1.08324	0.1913
C	-0.24567	-0.04577	0.1
H	-0.21117	-2.16648	-0.1826
H	-2.6634	-2.37315	-0.35982
H	-4.09435	-0.36403	-0.19367
H	-3.05424	1.85533	0.1532
H	-0.602	2.05529	0.33156
H	1.55935	1.01175	-0.37911
O	1.60973	2.30777	-1.18871
H	0.92771	2.03442	-1.82485

C	1.63778	0.5149	1.69423
H	2.70923	0.69092	1.76225
H	1.10865	1.42055	1.98463
H	1.3662	-0.28601	2.38201

Reactant complexes (RC) of the studied abstraction reactions by OH:

propyne + OH

C	-0.65942100	1.46949700	-0.00005800
H	-0.50984800	2.52173100	0.00064200
C	-0.85499800	0.28692600	0.00005100
C	-1.06362300	-1.15575200	0.00000400
H	-0.10188000	-1.66808000	0.00135400
H	-1.62276300	-1.45945200	0.88354500
H	-1.62044700	-1.45976300	-0.88489600
O	2.23449000	-0.23518900	0.00020300
H	1.44727100	0.34305300	-0.00224800

1-butyne + OH

C	1.70138400	-1.05155200	-0.27803100
H	2.68748100	-1.21523500	-0.63893400
C	0.58608000	-0.89699300	0.13471700
C	-0.77959500	-0.66207900	0.59961200
H	-0.74087500	0.01202700	1.45656600
H	-1.19608200	-1.60720200	0.95076300
O	0.58962800	2.15481200	0.09673800
H	1.12294800	1.34268800	0.00017600
C	-1.66400300	-0.06240300	-0.49631700
H	-1.70752900	-0.72344300	-1.35994000
H	-2.67449700	0.08983000	-0.12142000
H	-1.27166400	0.90100300	-0.82100500

1-pentyne + OH

C	1.88452	-1.01457	-0.64352

H	2.75684	-1.07154	-1.24804
C	0.90191	-0.97932	0.04349
C	-0.31219	-0.8981	0.85351
H	-0.17434	-0.11033	1.5984
H	-0.435	-1.83813	1.39309
O	1.04622	2.10864	0.41567
H	1.39005	1.25937	0.07707
C	-1.55918	-0.59664	0.01137
H	-1.68934	-1.39115	-0.72467
H	-2.42534	-0.62774	0.6734
C	-1.48299	0.75571	-0.68314
H	-2.38657	0.95254	-1.25835
H	-1.3621	1.56086	0.04365
H	-0.6364	0.79458	-1.37015

penta-1,4-diyne + OH

C	1.14590800	-1.71165500	-0.22343000
H	1.76690600	-2.48629100	-0.60334200
C	0.42410400	-0.85929200	0.20738900
C	-0.43443700	0.20440700	0.73524000
H	0.09178500	1.15673600	0.63349400
H	-0.59220500	0.03924400	1.80289200
O	2.55822100	1.36518100	-0.26900400
H	2.27513300	0.43718900	-0.37716800
C	-1.72824600	0.27665500	0.05520500
C	-2.78726400	0.34446000	-0.49428000
H	-3.72778600	0.40422300	-0.98458500

4-penten-1-yne + OH

C	-2.18970800	-0.28416800	-0.51762000
H	-2.96766100	-0.76294000	-1.06077900
C	-1.33245300	0.27611500	0.10408100

C	-0.25219000	0.91635300	0.84742200
H	-0.14662600	0.41211300	1.81088100
H	-0.52705100	1.95132800	1.06687500
O	0.91839000	-1.89517900	0.46679200
H	0.05246100	-1.71000000	0.06112700
C	1.07915300	0.88410800	0.14053800
C	1.28997800	0.45458700	-1.09376900
H	2.28324500	0.45423000	-1.51889500
H	1.91051000	1.24320800	0.73570800
H	0.47932400	0.09152500	-1.71316100

isopentyne + OH

C	-0.73148600	1.95214400	0.37212300
H	-1.26995000	2.82775200	0.64201400
C	-0.10406500	0.97573500	0.06851400
C	0.65119900	-0.23180800	-0.27783600
H	0.24993200	-0.59966900	-1.22536600
O	-2.48375300	-0.86468100	-0.46563600
H	-2.17987500	0.04462900	-0.28101600
C	2.13513400	0.09759500	-0.45836800
H	2.55497300	0.46647200	0.47741500
H	2.67936200	-0.79998600	-0.75057500
H	2.28003400	0.85877500	-1.22263400
C	0.44332700	-1.31458000	0.78538200
H	0.99009600	-2.21489700	0.50643200
H	0.81159900	-0.96929800	1.75149300
H	-0.61080300	-1.57083800	0.88842900

3-phenyl-1-propyne + OH

C	3.64475900	-0.64804200	-0.16247600
H	4.49228800	-1.22966900	-0.43063400
C	2.69648000	0.01412000	0.14559000

C	1.53823000	0.82006900	0.51500400
C	-0.91391700	0.68791900	0.99126300
C	-2.17117500	0.12246300	0.83685200
C	-2.32776100	-1.02376700	0.06920600
C	-1.21705900	-1.60318600	-0.52966100
C	0.04288900	-1.03999100	-0.36781900
C	0.20401200	0.11455600	0.38752700
H	-0.79770500	1.59242700	1.57523300
H	-3.02863300	0.58376300	1.30689600
H	-3.30676400	-1.46478300	-0.05727800
H	-1.32941600	-2.50031600	-1.12341100
H	0.90728600	-1.49325500	-0.83399600
H	1.50900100	1.70937700	-0.12111000
H	1.66353500	1.17389700	1.53998800
O	-0.98095600	1.98659900	-1.44060500
H	-1.24069000	1.07092200	-1.64376500

but-3-yn-2-ylbenzene + OH

C	2.94164100	0.79777500	-0.83363700
H	3.64178800	1.43445300	-1.31670000
C	2.16072500	0.08301900	-0.27133700
C	1.19453300	-0.77999500	0.41068300
C	-0.62511800	0.07163100	-1.13956200
C	-1.94752900	0.40913700	-1.39298400
C	-2.89579800	0.32166900	-0.38369800
C	-2.51439900	-0.10389100	0.88103300
C	-1.19232900	-0.44032700	1.13230500
C	-0.23646700	-0.35808400	0.12550900
H	0.11676100	0.14965600	-1.92481200
H	-2.23635100	0.74352700	-2.38024100
H	-3.92502800	0.58868400	-0.58000200

H	-3.24548800	-0.16701100	1.67554900
H	-0.89407300	-0.76059000	2.12310300
H	1.36615300	-0.66293300	1.48276900
O	1.29550200	2.24160700	1.16679400
H	0.64794300	2.08368300	0.45819900
C	1.41192900	-2.25232100	0.02733200
H	2.42803000	-2.56366600	0.26300700
H	0.70919800	-2.88202800	0.57131400
H	1.24392700	-2.38830300	-1.04039600

Product complexes (PC) of the studied abstraction reactions by OH:

propyne + OH

C	1.21596100	-1.22134000	-0.00587400
H	1.41936700	-2.26419500	-0.00693600
C	1.01001400	-0.02389300	-0.00373900
C	0.74576100	1.32598200	0.00268500
H	-2.13331100	-0.23648200	-0.88428600
H	-0.27363000	1.66780700	0.10079400
H	1.54939600	2.04071900	-0.08160300
O	-2.13102500	-0.13522300	0.07007500
H	-1.34403900	-0.61056000	0.35300100

1-butyne + OH

C	-0.71420100	1.89880300	-0.23664300
H	-1.45320500	2.59889900	-0.54095400
C	0.16057400	1.13166300	0.11069500
C	1.13654200	0.22727600	0.48156400
H	-1.81077700	-1.24573900	0.97584300
H	1.60421100	0.35434900	1.44812800
O	-1.75507100	-1.40920400	0.03172000
H	-1.54872700	-0.54466400	-0.33697600

C	1.50022900	-0.94540400	-0.36466700
H	2.54609300	-1.21656200	-0.22867900
H	0.88448700	-1.81008900	-0.09889800
H	1.31962000	-0.73658600	-1.41791100

1-pentyne + OH

C	1.83039	-1.18844	-0.53892
H	2.67633	-1.36574	-1.15692
C	0.85259	-1.0335	0.16524
C	-0.25833	-0.81808	0.95548
H	1.14495	2.16463	1.01258
H	-0.21557	-1.15187	1.98384
O	0.94811	2.13635	0.07384
H	1.28313	1.27956	-0.21055
C	-1.4858	-0.10733	0.48081
H	-2.35992	-0.64234	0.86176
H	-1.51207	0.88246	0.95109
C	-1.57411	0.05474	-1.02959
H	-2.51171	0.53407	-1.30578
H	-0.75853	0.67615	-1.39525
H	-1.52001	-0.91212	-1.5296

penta-1,4-diyne + OH

C	0.96829600	-1.86901900	-0.02347300
H	1.53047600	-2.77003400	-0.06454800
C	0.30296000	-0.86083500	0.02397400
C	-0.42782500	0.32454100	0.08555400
H	2.76066500	1.23261100	-0.90559300
H	0.12166800	1.25046800	0.20211700
O	2.64681400	1.20857700	0.04712400
H	2.45633600	0.28567900	0.24127100
C	-1.81574900	0.34310000	0.01045600

C	-3.02129400	0.38209100	-0.05379100
H	-4.08198800	0.41338700	-0.10656100

4-penten-1-yne + OH

C	-1.90101700	0.99670500	-0.50491300
H	-2.85189500	0.94508400	-0.97726400
C	-0.82151700	1.07309500	0.02871400
C	0.43288700	1.14440100	0.64984500
H	0.02063700	-2.01371100	0.68142800
H	0.52444400	1.79626600	1.50721500
O	-0.87518400	-2.19726200	0.38905000
H	-1.25886600	-1.32477100	0.25910100
C	1.55336000	0.41772200	0.21157000
C	1.56355700	-0.43429600	-0.85012500
H	2.46396000	-0.95878000	-1.13207600
H	2.46805200	0.56038200	0.77374500
H	0.67151600	-0.61213000	-1.43509200

isopentyne + OH

C	-1.87901700	1.35105000	-0.00780400
H	-2.87656000	1.71609000	-0.00393900
C	-0.71857700	0.99276100	-0.01098300
C	0.58709300	0.53354100	-0.01041800
H	-1.20001500	-2.62570200	-0.65656400
O	-0.80732100	-2.19169500	0.10323000
H	-1.24038300	-1.33275300	0.14906200
C	1.25836600	0.15687300	-1.28945200
H	2.28117700	0.53922700	-1.31690100
H	1.31647700	-0.93460500	-1.35881000
H	0.71799800	0.52999200	-2.15593100
C	1.26983100	0.19404700	1.27326300
H	1.27478500	-0.89366000	1.40414500

H	2.31015800	0.52563400	1.25645700
H	0.76874900	0.63969800	2.12901100

3-phenyl-1-propyne + OH

C	-3.35784000	-1.38421200	0.07867400
H	-4.17029100	-2.00179200	0.37414300
C	-2.43814900	-0.67870200	-0.26510000
C	-1.38266900	0.13790400	-0.65786600
C	0.98395000	0.76698100	-0.77949700
C	2.31500500	0.53045300	-0.49620900
C	2.68944200	-0.61058500	0.20698500
C	1.71679000	-1.51393900	0.62573900
C	0.38346100	-1.28506000	0.34977400
C	-0.01212200	-0.13639700	-0.35996300
H	0.68781600	1.65702000	-1.32052100
H	3.06720700	1.23474800	-0.82439900
H	3.73178600	-0.79473500	0.42681400
H	2.00603900	-2.40191500	1.17120200
H	-0.37383800	-1.98646700	0.67401200
H	-1.63010700	2.43124400	1.10059600
H	-1.61586300	1.04097500	-1.20629000
O	-0.87079500	2.93864000	0.80433100
H	-0.12360100	2.35314000	0.95457800

but-3-yn-2-ylbenzene + OH

C	2.92842800	-1.75921700	-0.09998400
H	3.63052000	-2.50491300	-0.38252000
C	2.13129000	-0.91433700	0.23616000
C	1.23289600	0.08876900	0.60708000
C	-0.70101000	-1.29706900	-0.10784700
C	-2.05484800	-1.44669600	-0.33347000
C	-2.93397700	-0.40069000	-0.06974900

C	-2.43923000	0.79988700	0.42472500
C	-1.08440000	0.95806400	0.65646900
C	-0.18131700	-0.08923500	0.39531000
H	-0.02152100	-2.11259600	-0.31494500
H	-2.43178700	-2.38436800	-0.71873000
H	-3.99318400	-0.52046300	-0.24951000
H	-3.11455400	1.61951500	0.62941900
H	-0.71714700	1.90210800	1.03299700
H	1.46783500	0.82901500	-2.02194700
O	0.91378600	1.60551500	-2.13894100
H	0.04834100	1.32258200	-1.83061300
C	1.78062800	1.37443400	1.14645700
H	2.85231500	1.29834300	1.30583200
H	1.59276700	2.18548200	0.43718800
H	1.30536100	1.63772700	2.09345300
